# Curcumin Attenuates Titanium Particle-Induced Inflammation by Regulating Macrophage Polarization *In Vitro* and *In Vivo*

**DOI:** 10.3389/fimmu.2017.00055

**Published:** 2017-01-31

**Authors:** Bin Li, Yan Hu, Yaochao Zhao, Mengqi Cheng, Hui Qin, Tao Cheng, Qiaojie Wang, Xiaochun Peng, Xianlong Zhang

**Affiliations:** ^1^Department of Orthopedics, Shanghai Sixth People’ Hospital, Shanghai Jiao Tong University, Shanghai, China

**Keywords:** curcumin, osteolysis, titanium particle, inflammation, macrophage polarization

## Abstract

Periprosthetic inflammatory osteolysis and subsequent aseptic loosening are commonly observed in total joint arthroplasty. Other than revision surgery, few approved treatments are available for this complication. Wear particle-induced inflammation and macrophage polarization state play critical roles in periprosthetic osteolysis. We investigated the effects of curcumin, a polyphenol extracted from *Curcuma longa*, on titanium (Ti) particle-induced inflammation and macrophage polarization *in vitro* using the murine cell line RAW 264.7 and *in vivo* using a murine air pouch model. The expression of specific macrophage markers was qualitatively analyzed by immunofluorescence (inducible nitric oxide synthase and CD206) and quantitatively analyzed by flow cytometry (CCR7 and CD206), representing M1 and M2 macrophages, respectively. Our results show that curcumin induced a higher percentage of M2 macrophages together with a higher concentration of anti-inflammatory cytokine IL-10, and a lower percentage of M1 macrophages with a lower concentration of pro-inflammatory cytokines (TNF-α and IL-6). The genes encoding CD86 (M1) and CD163 (M2), two additional markers, were shifted by curcumin toward an M2 phenotype. C57BL/J6 mice were injected with air and Ti particles to establish an air pouch model. Curcumin reduced cell infiltration in the pouch membrane and decreased membrane thickness. The analysis of exudates obtained from pouches demonstrated that the effects of curcumin on macrophage polarization and cytokine production were similar to those observed *in vitro*. These results prove that curcumin suppresses Ti particle-induced inflammation by regulating macrophage polarization. Thus, curcumin could be developed as a new therapeutic candidate for the prevention and treatment of inflammatory osteolysis and aseptic loosening.

## Introduction

Total joint arthroplasty is a highly successful surgical technique used to relieve pain and improve both movement and the quality of life for patients with severe joint diseases (1). However, inflammatory osteolysis with subsequent aseptic loosening remains a major complication that limits the long-term survivorship of prosthetic joints. Despite the serious consequences of this complication, few non-operative treatments have emerged. Thus, it is essential to develop new strategies and drugs to treat inflammatory osteolysis and aseptic loosening.

The local inflammation induced by wear particles, which are generated at the interface of the implant and the bone, is critically important for understanding the underlying cause of osteolysis and consequent aseptic loosening (2). Macrophages play an important role in the particle-induced inflammatory cascade. After stimulated by wear particles, macrophages increase the expression of pro-inflammatory factors, including cytokines, chemokines, and other substances (3, 4). One current hypothesis on macrophage polarization and plasticity has aroused interest in the pathogenesis of wear particle recognition and inflammatory osteolysis (5, 6). The concept of “classically” and “alternatively” activated macrophages, or M1 and M2 macrophages, is based on the Th1/Th2 polarization paradigm of T lymphocytes (7). M1 macrophages are induced by microbial products or pro-inflammatory cytokines such as lipopolysaccharide (LPS) and interferon gamma (IFN-γ). M1 macrophages exhibit increased phagocytic activity and increased production of pro-inflammatory cytokines such as interleukin-6 (IL-6), tumor necrosis factor alpha (TNF-α), and inducible nitric oxide synthase (iNOS) to promote inflammation. By contrast, M2 macrophages are induced by IL-4 or IL-13 and represent a phenotype that contributes to the resolution of inflammation and wound healing by producing various anti-inflammatory cytokines such as IL-10 and arginase-1 (ARG-1) (8). It has been recognized that the existence of wear particles initiates the differentiation and activation of macrophages to a classic M1 phenotype that promotes local inflammation (9). Thus, modulation of the macrophage activation state would appear to be a novel strategy for attenuating wear particle-induced inflammation (10). Indeed, evidence is emerging that the modulation of macrophages from an M1 phenotype to an M2 phenotype is a viable method to reduce particle-induced inflammation (11–13).

Curcumin, a major bioactive compound derived from turmeric (*Curcuma longa*), exhibits antioxidant, anti-inflammatory, antimicrobial, and anticancer activities, as well as excellent safety (14). Curcumin is effective against various inflammation-related diseases such as bronchial asthma, chronic cutaneous complications, gingivitis, and glomerulonephritis (15, 16). A recent study revealed that curcumin suppresses inflammation by shifting macrophages from an M1 to an M2 polarization phenotype (17). Another study reported that curcumin treatment alleviates inflammation and induces M2 polarization in macrophages in an experimental autoimmune myocarditis model by the secretion of IL-4 and/or IL-13 (18). The molecular analysis of curcumin-induced macrophage polarization demonstrated that curcumin inhibits the M1 macrophage phenotype by inhibiting nuclear factor kappa-light-chain-enhancer of activated B cells (NF-κB) and by activating IκBα, and by promoting the M2 phenotype through the activation of proliferator-activated receptor gamma (PPAR-γ) (19). Toll-like receptor 4 (TLR4) and its signaling pathway are also inhibited by curcumin *via* the regulation of macrophage polarization (20). On the basis of these studies, we speculated that curcumin attenuates titanium (Ti) particle-induced inflammation by regulating macrophage polarization. In this study, we tested this hypothesis *in vitro* using the murine macrophage cell line RAW264.7 and *in vivo* using a murine air pouch model.

## Materials and Methods

### Particles

Commercially pure Ti particles were purchased from Johnson Matthey Chemicals (catalog #00681, Ward Hill, MA, USA). The average diameter of the particles was 2.9 µm according to the manufacturer’s certificate of analysis. The particles were baked at 180°C for 6 h and then washed in 70% ethanol for 48 h to remove endotoxins, as previously described (21). The endotoxin level of the particle was determined with a Limulus Amebocyte Lysate Assay (Biowhittaker, Walkersville, MD, USA) and only endotoxin-free particles were used in this study.

### Cell Culture

Murine macrophage RAW 264.7 cells (American Type Culture Collection, Cell Bank of Chinese Academy of Sciences, China) were cultured in Dulbecco’s Modified Eagle’s Medium (Hyclone, USA) containing 10% fetal bovine serum (FBS; Gibco, USA) and 1% antibiotics (100 U/mL penicillin-G and 100 pg/mL streptomycin) in a humidified atmosphere of 5% CO_2_ at 37°C. The cells were passaged at approximately 80% confluence by scraping and only early passages (p3–5) were used. The RAW cells were plated onto different cell culture plates before stimulated with or without Ti particles (0.1 mg/mL) or stimulated with a combination of Ti particles (0.1 mg/mL) and different concentrations of curcumin (0, 6.25, and 25 nM). These treatments were designated as Control, Ti, Ti + Cur 6.25, and Ti + Cur 25. Images of RAW cells were obtained on a light microscope (Leica).

### CCK-8 Assay

The proliferation of RAW cells was evaluated using a Cell Counting Kit-8 Assay (CCK-8, Dojindo, Japan). Cells were plated onto 24-well plates at a density of 1.0 × 10^5^ cells per well. After 1 and 4 days of culture, fresh medium containing 10% CCK-8 solution was added to each well and incubated at 37°C for 4 h. After incubation, 100 µL supernatant was added to a new 96-well plate. Absorbance was measured on a microplate reader at a wavelength of 450 nm.

### Immunofluorescent Staining

RAW cells were seeded onto 24-well plates (1 × 10^5^ cells/well). After culturing for 1 and 4 days, 4% paraformaldehyde in phosphate-buffered saline (PBS) was used to fix the cells for 15 min at room temperature. Then the cells were washed three times in PBS containing 0.1% Triton-X for permeabilization. Non-specific binding sites were blocked with 10% FBS in PBS for 1 h. Primary monoclonal antibodies for iNOS (Novus Biologicals) and CD206 (1:50) (AbCam) were incubated in PBS containing 1% FBS at 4°C overnight. Cells were washed three times in PBS. Donkey anti-rabbit Alexa Fluor 488 (1:200) and donkey anti-mouse Alexa Fluor 594 (1:200) (AbCam) were incubated at room temperature for 2 h as secondary antibodies. Cell nuclei were stained with DAPI for 15 min. Then the cells were washed three times in PBS. Images were collected on a fluorescence microscope (Leica).

### Flow Cytometry

RAW cells were seeded onto 6-well plates (5 × 10^5^ cells/well). Macrophage cell subpopulation markers CCR7 (M1) and CD206 (M2) were analyzed by flow cytometry to evaluate the different phenotypes. After 1 and 4 days of culture, cells were trypsinized, scraped from the plates, centrifuged, and resuspended in 1% bovine serum albumin (BSA) for 30 min at ambient temperature to block non-specific antigens. Then the cells were incubated with allophycocyanin (APC)-conjugated CCR7 and phycoerythrin (PE)-conjugated CD206 for 30 min at ambient temperature. The isotype controls used were APC-conjugated Armenian hamster IgG and PE-conjugated rat IgG2a,κ. All antibodies used for flow cytometry were purchased from eBioscience. After washing twice with PBS, cells were resuspended in 1% BSA and analyzed on a Guava flow cytometer (Millipore, USA). Data were analyzed using guavaSoft 3.1.1 software.

### ELISA

After 1 and 4 days of culture, the culture medium was aspirated and centrifuged at 2,500 rpm for 10 min. The supernatants were used for subsequent analyses. The concentrations of cytokines TNF-α, IL-6, and IL-10 were determined using ELISA kits (Anogen, Canada) following the manufacturer’s recommendations.

### PCR

The gene expression of two specific macrophage markers, CD86 and CD163, was analyzed by real-time PCR. RAW cells were seeded onto 6-well plates (5 × 10^5^ cells/well) and cultured for 1 and 4 days. RNA was extracted using TRIzol reagent (Invitrogen, USA) after washing the cells twice with PBS. Total RNA (300 ng) was used to synthesize complementary DNA using the PrimeScript RT Reagent Kit (Takara, Japan) following the manufacturer’s instructions. SYBR Premix Ex TaqII (Takara) was used for detection, and the expression of the target mRNAs were assayed on a Bio-Rad C1000. The mean cycle threshold (Ct) value of the housekeeping gene GAPDH was used to normalize the target genes. The 2^−ΔΔCt^ method was used to determine relative gene expression. The following forward and reverse primer sequences were used: for CD86, 5′-TGC TCA TCA TTG TAT GTC AC-3′, and 5′-GTC TCT CTG TCA GCG TTA CT-3′, and for CD163, 5′-TCA GCG ACT TAC AGT TTC CTC-3′, and 5′-GCC TTT GAA TCC ATC TCT TG-3′.

### Air Pouch Model

Animal care and use were in accordance with the guidelines established by the Administration of Affair Concerning Laboratory Animals for Shanghai Jiao Tong University, the National Institutes of Health Guide for Care and Use of Laboratory Animals (GB14925-2010), and the Regulations for the Administration of Affairs Concerning Experimental Animals (China, 2014). All animal experiments described in this study were approved by the Animal Care and Experiment Committee of Shanghai Sixth People’s Hospital affiliated with Shanghai Jiao Tong University. Six male C57BL/6 mice weighing approximately 25 g (8 weeks old) were used for each experimental group. Air pouches were generated according to the method of Sedgwick et al. (22). After air pouch formation, pouches were injected with 0.5 mL PBS alone (Control) or 0.5 mL PBS containing Ti particulates with or without curcumin (6.25 or 25 nM). The mice received repeated curcumin injections daily (6.25 or 25 nM, 0.5 mL) until sacrifice. After 1 and 4 days of the initial injection, the mice were anesthetized by intraperitoneal injection of chloral hydrate (350 mg/kg). Then inflammatory exudates were harvested by collecting the lavage fluid after washing the air pouch cavities with 2 mL PBS. The mice were sacrificed on day 7, and the pouch membranes were fixed in formalin for histological analysis.

### Histological Analysis

The fixed pouch membranes were processed and embedded in paraffin wax and cut into 4-µm sections. These sections were stained with hematoxylin and eosin (HE) and examined under a light microscope (Leica) to observe the morphology of the pouch membrane. Pouch membrane thickness and the total number of cells were analyzed based on six different random locations using Image-Pro Plus software as previously described (23).

### Flow Cytometric Analysis of the Inflammatory Exudates

The inflammatory exudates were obtained and analyzed by flow cytometry according to the method described by Vasconcelos et al. (24). Exudates were filtered through a 40-µm nylon mesh (BD Biosciences) to remove cell clumps and impurities at 1,200 rpm for 5 min at 4°C. Cell pellets were resuspended in 1 mL 1% BSA/PBS and supernatants discarded. Cell suspensions were preincubated for 30 min at room temperature to block non-specific binding sites. Then cells were incubated with fluorescein isothiocyanate (FITC)-conjugated anti-mouse F4/80, APC-conjugated CCR7, and PE-conjugated CD206 for 30 min at 37°C at a final volume of 100 µL in the dark. FITC-conjugated rat IgG2a,κ, APC-conjugated rat IgG2a,κ, and 229 PE-conjugated rat IgG2a,κ were used as isotype controls. All antibodies used for flow cytometry were purchased from eBioscience. After washing twice in PBS, the cells were resuspended in 1% BSA and processed on a Guava flow cytometer (Millipore, USA). Data were analyzed using guavaSoft 3.1.1 software as previously described.

### Cytokine Analysis

Exudates were centrifuged at 2,500 rpm for 15 min, and the supernatants were collected. The concentrations of TNF-α, IL-6, and IL-10 in the inflammatory exudates were determined using ELISA kits (Anogen, Canada) according to the manufacturer’s instructions.

### Statistical Analysis

SPSS 18.0 software (SPSS, Chicago, IL, USA) was used to analyze the data. Data were analyzed using one-way analysis of variance (ANOVA) with S-N-K *post hoc t*-tests. Data are shown as the means ± SD The level of significance was set to *p* < 0.05.

## Results

### Cell Morphology and Proliferation

**Figure 1:**
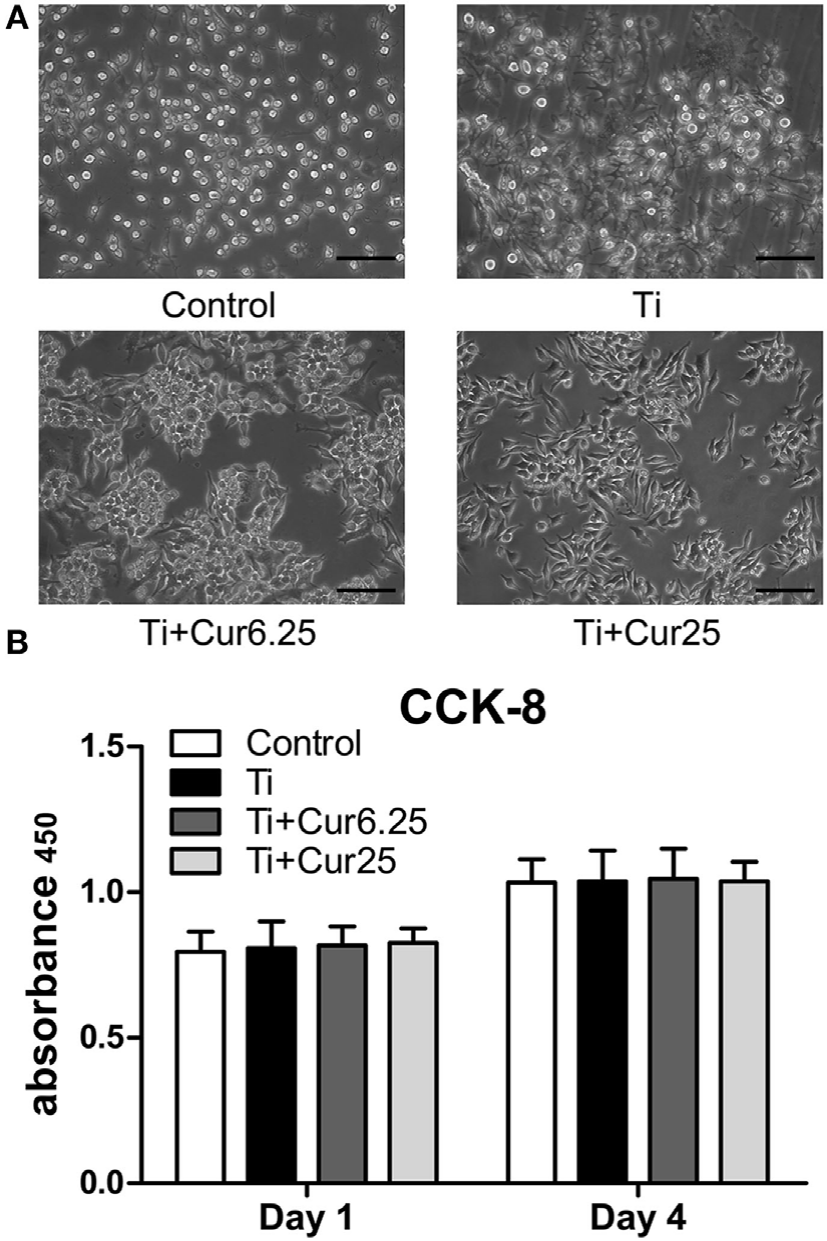
**(A)** Morphology of RAW cells obtained by a light microscope. Scale bar: 100 µm. **(B)** Cell proliferation was evaluated by CCK-8 after 1 and 4 days of culture (*p < 0.05; **p < 0.01).

The results from the morphological analysis are presented in Figure 1A. The majority of RAW cells in the control group were small and round without stimulation by Ti particulates. In the Ti group, the cells were flat and showed many synaptic structures. The number of cone-shaped cells was increased in groups receiving curcumin treatment. Curcumin alone did not induce morphological changes in the RAW cells when compared with control group in preliminary experiments. The subsequent studies focused on the effects of curcumin on Ti particle-induced changes in the RAW cells. Results from the CCK-8 assay are shown in Figure 1B. There were no statistically significant differences between the four groups, either at day 1 or day 4.

### Immunofluorescent Staining

**Figure 2:**
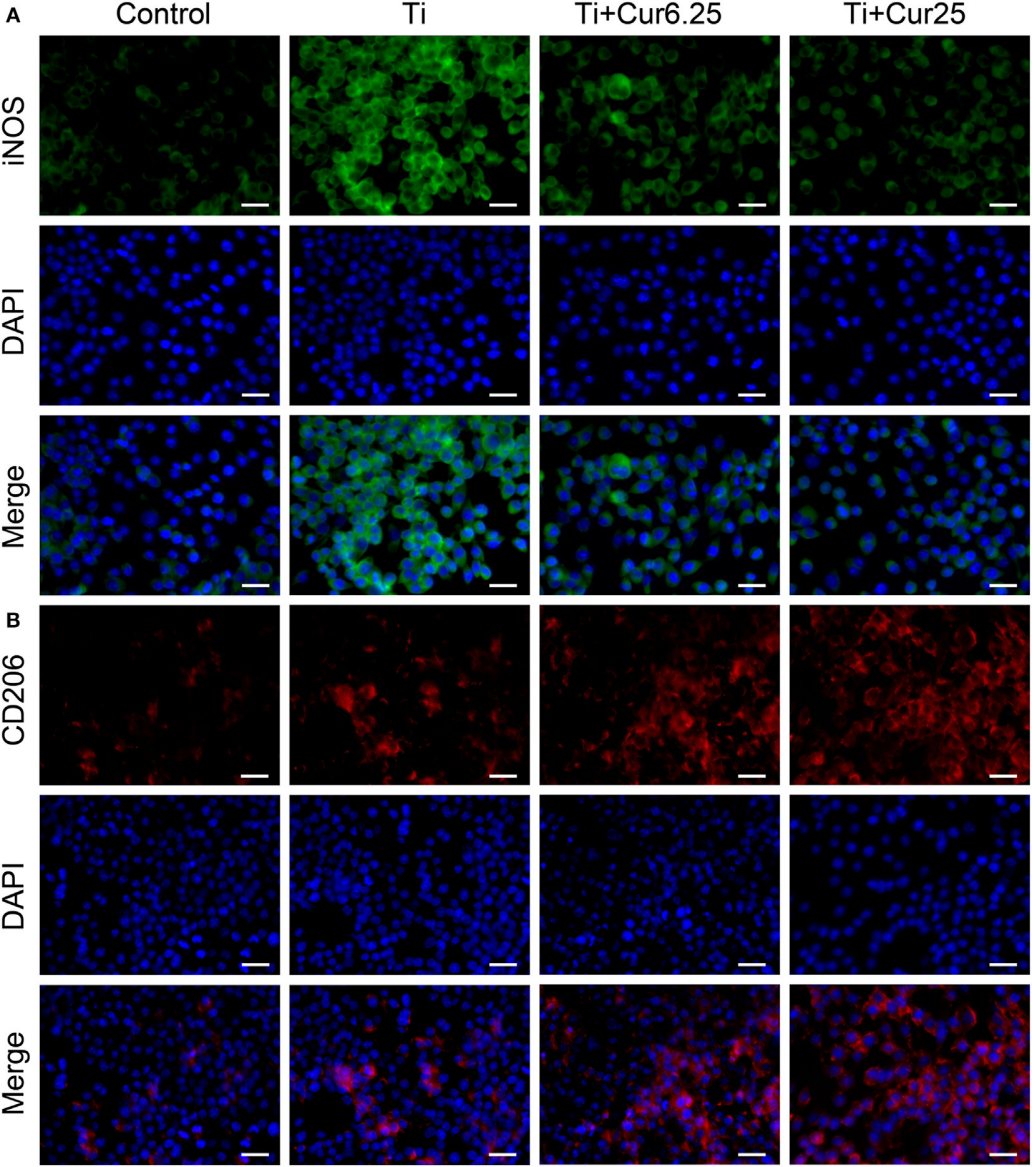
**Immunoflourescent staining of the RAW cells**. **(A)** Inducible nitric oxide synthase was in green fluorophore, represent M1 macrophages; **(B)** CD206 was in red fluorophore, represent M2 macrophages; nuclei stained with DAPI (blue fluorophore), scale bar: 25 µm.

Cells were stained with iNOS (an M1 marker), CD206 (an M2 marker), and DAPI (nucleus). As shown in Figure 2A, the expression of iNOS in the different groups showed the following trend: Ti > Ti + Cur 6.25 > Ti + Cur 25 > Control. This shows that the curcumin-treated groups had a lower percentage of M1 macrophages than the Ti group. By contrast, the trend of CD206 expression was: Control < Ti < Ti + Cur 6.25 < Ti + Cur 25 (Figure 2B). This demonstrates a higher percentage of M2 macrophages in the curcumin-treated groups relative to the Ti group. The control group had the lowest expression of both iNOS and CD206.

### Flow Cytometry

**Figure 3:**
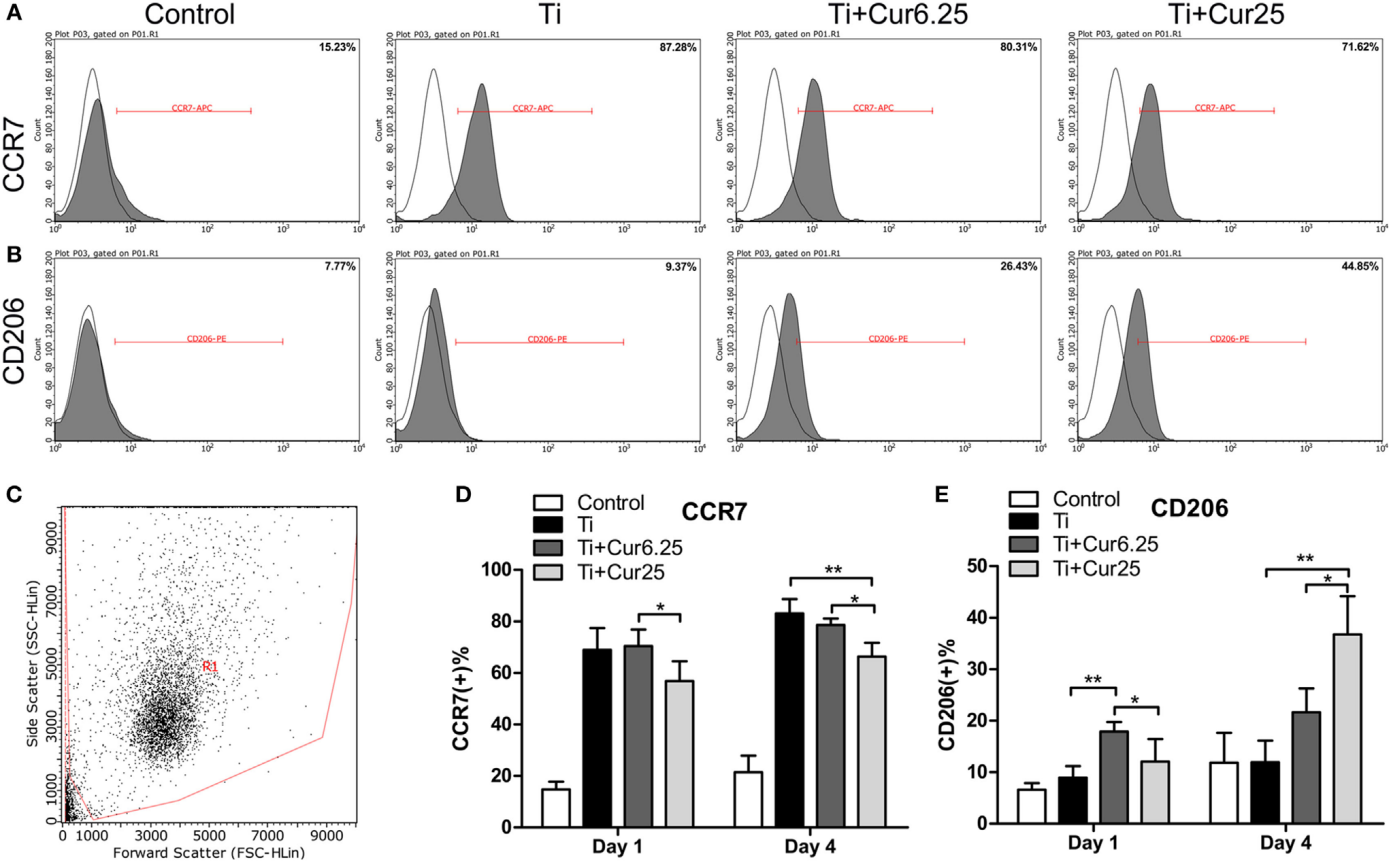
**(A,B)** Representative histograms of flow cytometric results (day 4), percentage of CCR7 positive or CD206 positive cells, representing M1 or M2 macrophages, respectively. **(C)** RAW cells were gated based on forward scatter and side scatter (region R1). **(D,E)** statistical results of CCR7 positive (M1) or CD206 positive (M2) macrophages (*p < 0.05; **p < 0.01).

The flow cytometry results are shown in Figure 3. The histograms of CCR7 (an M1 marker) and CD206 (an M2 marker) from a representative experiment are presented in Figures 3A,B. The dot plot in Figure 3C shows the forward scatter (FSC) and side scatter (SSC) of RAW cells. The statistical results from flow cytometry are presented in Figures 3D,E. The curcumin-treated groups contained a lower percentage of CCR7-positive cells (M1) and a higher percentage of CD206-positive cells (M2) than the Ti group, which was more obvious at day 4.

### Cytokine Production and Gene Expression *In Vitro*

**Figure 4:**
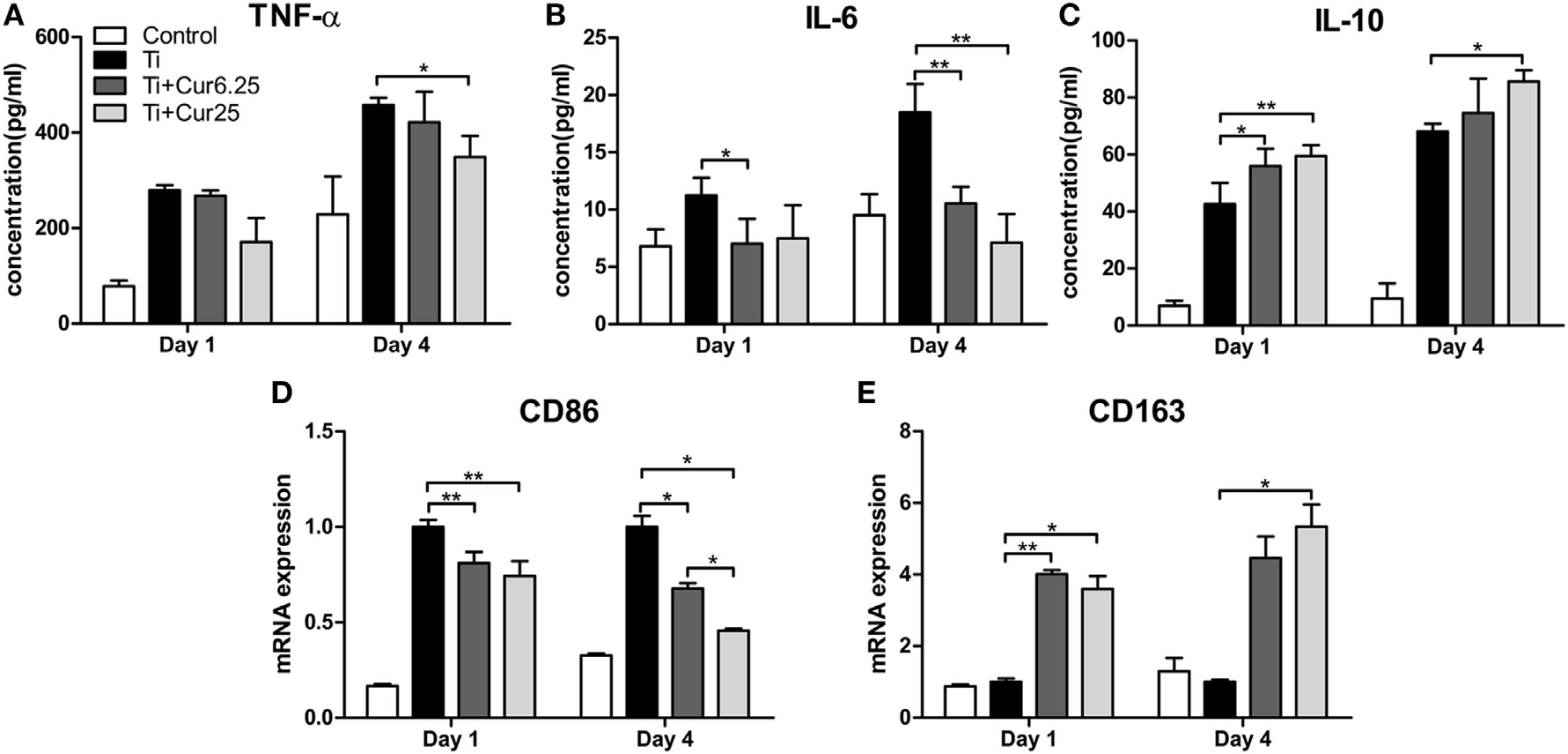
**ELISA results of cytokine production by RAW cells: (A) TNF-α; (B) IL-6; (C) IL-10**. **(D)** Gene expression of M1 marker CD86. **(E)** Gene expression of M2 marker CD163 (*p < 0.05; **p < 0.01).

The cytokine levels determined by ELISA and the gene expression levels of the two macrophage phenotype markers determined by PCR are depicted in Figure 4. The curcumin-treated groups expressed lower concentrations of TNF-α than the Ti group, albeit the difference was only statistically significant at 4 days of culture (Figure 4A). A lower concentration of IL-6 was detected in the curcumin-treated groups both at days 1 and 4 (Figure 4B). The anti-inflammatory cytokine, IL-10, was significantly upregulated in the curcumin-treated groups relative to the Ti group both at days 1 and 4 (Figure 4C). The gene expression of CD86, representing M1 macrophages, was downregulated in the curcumin-treated groups both at days 1 and 4 of culture compared to the Ti and control groups (Figure 4D). By contrast, the M2 macrophage marker gene CD163 was upregulated in the curcumin-treated groups at both time points (Figure 4E).

### Histological Analysis

**Figure 5:**
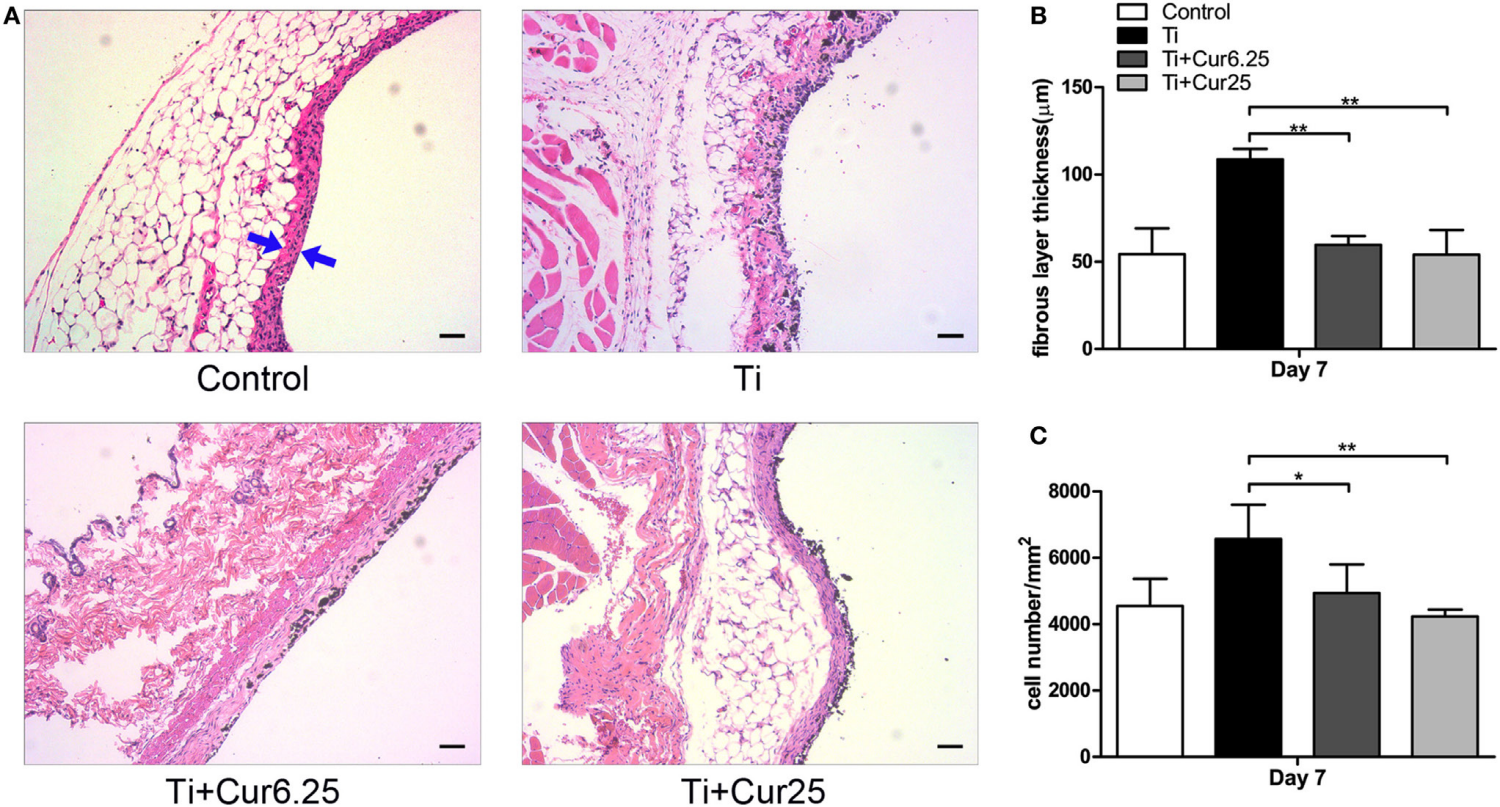
**(A)** Histological appearance of the pouch membrane. Scale bar: 50 µm. **(B)** Statistical analysis of fibrous layer thickness. **(C)** Cell infiltration of pouch membrane. Blue arrows: fibrous layer (*p < 0.05; **p < 0.01).

Air pouch tissues were stained with HE to evaluate the histological appearance. Examination of the tissue sections demonstrated that pouches injected with Ti particulates provoked pronounced inflammatory responses, presenting as increased thickness and cellular infiltration of the pouch membranes compared to pouches injected with PBS alone. In contrast to the Ti group, curcumin ameliorated the inflammation by reducing the pouch membrane thickness and cellular infiltration (Figure 5A). The quantitative analyses were consistent with the general observations, as shown in Figures 5B,C.

### Flow Cytometric Analysis of the Inflammatory Exudates

**Figure 6:**
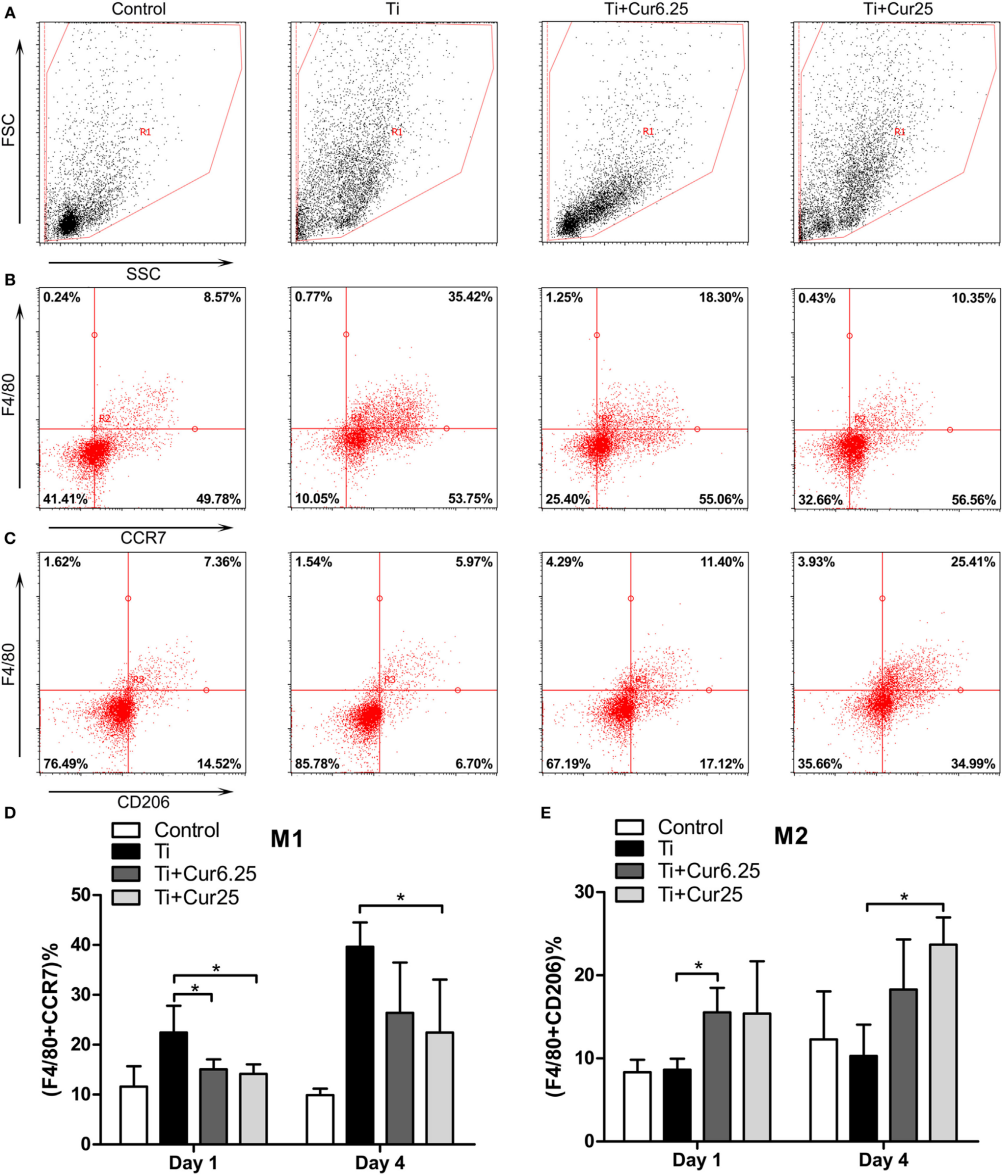
**Flow cytometric analysis of cells obtained from the inflammatory exudates**. Representative dot plots (day 4) showing: **(A)** forward scatter (FSC) and side scatter (SSC), cells were gated based on FSC and SSC (region R1); **(B)** CCR7 and F4/80 double-positive cells, representing M1 macrophages; **(C)** CD206 and F4/80 double-positive cells, representing M2 macrophages. **(D)** Percentage of M1 macrophages; **(E)** percentage of M2 macrophages (*p < 0.05; **p < 0.01).

The inflammatory exudates from the air pouches were collected at days 1 and 4 after injection with Ti particulates. Then the different macrophage phenotypes in the exudates were analyzed by flow cytometry. The macrophages were identified by F4/80 and two specific markers, CCR7 (M1) and CD206 (M2). Representative dot plots of the different experimental groups are displayed in Figure 6. The FSC and SSC of the cells in the exudates are shown in Figure 6A. The use of specific macrophage phenotype markers showed that the injection of curcumin caused an increase in the number of F4/80 and CD206 double-positive cells and a decrease in the number of F4/80 and CCR7 double-positive cells compared to the Ti and control groups (Figures 6B,C). The trend was more obvious in the Ti + Cur 25 group than in the Ti + Cur 6.25 group. This difference was statistically significant at both time points, as shown in Figure 6D.

### Cytokine Production *In Vivo*

**Figure 7:**
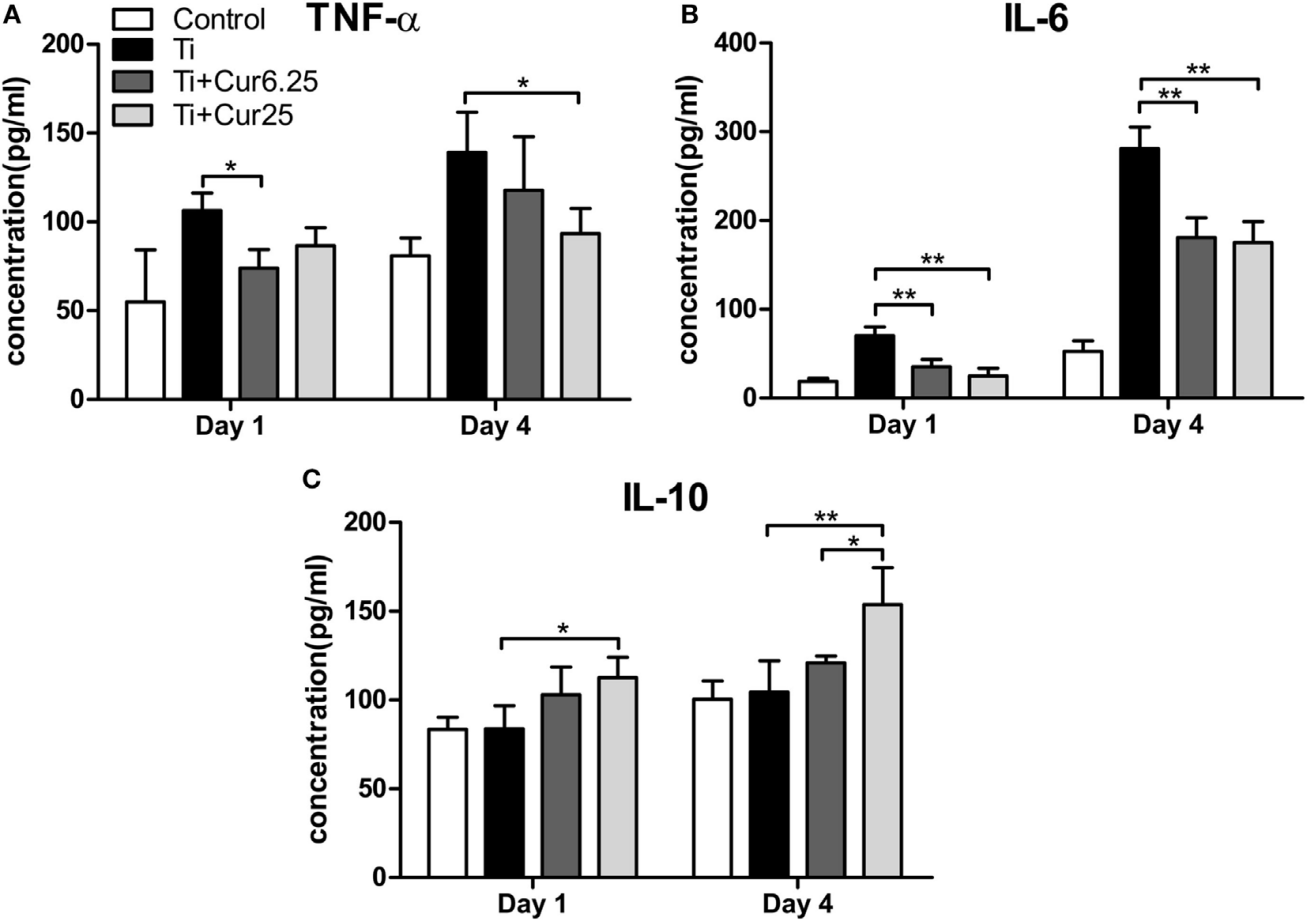
**Cytokine productions in inflammatory exudates were evaluated by ELISA**. **(A)** TNF-α, **(B)** IL-6, and **(C)** IL-10 (*p < 0.05; **p < 0.01).

The levels of pro-inflammatory cytokines (TNF-α and IL-6) and anti-inflammatory cytokine IL-10 are presented in Figure 7. Injection of curcumin reduced the expression of TNF-α both at days 1 and 4 after injection with Ti particulates (Figure 7A). The decrease in IL-6 was more obvious and there was a statistically significant difference between the curcumin-injected groups and the Ti group both at days 1 and 4 after injection (Figure 7B). The anti-inflammatory cytokine, IL-10, was upregulated in the exudates injected with curcumin (Figure 7C). Taken together, these data indicate that the injection of curcumin-induced higher levels of anti-inflammatory cytokines and reduced the production of pro-inflammatory cytokines.

## Discussion

Although many researchers have made several attempts to develop new drugs to prevent or treat inflammatory osteolysis with subsequent aseptic loosening, few non-operative methods are presently available. Most patients at the end stage of aseptic loosening have to undergo revision surgery, which increases medical costs and extends the hospitalization time. Wear particle-induced inflammation is believed to play a critical role in the pathogenesis of osteolysis and aseptic loosening. The macrophage polarization state may also affect the inflammatory cascade. The current study described the potential of curcumin to attenuate Ti particle-induced inflammation *in vitro* using a murine macrophage cell line, RAW 264.7, and *in vivo* using a murine air pouch model by regulating macrophage polarization to an M2 phenotype.

Commercially available Ti particles are widely used to mimic wear particles generated at the periprosthetic tissue (21, 23). It is necessary to use only endotoxin-free particles because the inflammation induced by endotoxins has a different pattern compared to that induced by wear particles in terms of cellular and cytokine profiles (25). The morphology of RAW 264.7 cells revealed that Ti particles induced obvious M1 macrophage differentiation and activation characterized by the spreading and growing of many synaptic structures. Curcumin significantly increased the number of M2 cone-shaped macrophages and reduced the differentiation of M1 macrophages, particularly at higher concentrations. The results of the CCK-8 assay indicated that Ti particles and curcumin have no obvious toxic influence on the proliferation of RAW 264.7 cells. The RAW cells were stained with macrophage specific markers iNOS and CD206 to identify M1 and M2 macrophage phenotypes. Cells cultured with curcumin presented higher numbers of M2 macrophages compared to cells cultured with Ti particles. It appeared that the inflammation was attenuated by curcumin, as iNOS is not only a marker for M1 macrophages but is also known as an indicator for the extent of inflammation (26). The morphological and immunostaining results were qualitative, as macrophages presented a continuous spectrum between the different phenotypes. To quantify these observations, flow cytometry was employed to assess the percentages of different macrophage phenotypes. The results were consistent with previous observations that curcumin induced a higher percentage of M2 macrophages and the trend was more significant after 4 days of culture. Surprisingly, it appears that Ti particles induce a slightly higher percentage of M2 macrophages than the Control group, although the difference did not reach statistical significance; this may be the feedback of M1 macrophage activation but the exact mechanism requires further investigation. The cytokine levels of different macrophage polarization phenotypes were analyzed by ELISA. Curcumin decreased the secretion of two pro-inflammatory cytokines (TNF and IL-6) and increased the secretion of anti-inflammatory cytokine IL-10, which is characteristic of a downregulation of inflammation and a shift toward M2 macrophage activation (8). We have also tried to test the concentration of another anti-inflammatory cytokine, IL-4, but the concentrations were quite low, being below the recommended detection range (7.8 pg/mL). One possible explanation for this is that IL-4 is mainly secreted by Th2 cells as reported by many papers. At the genetic level, the expression of two additional specific macrophage markers, CD86 (M1) and CD163 (M2) were analyzed by PCR to further verify the effects of curcumin on macrophage polarization. The trend was consistent with other markers, as previously described. In general, the *in vitro* results indicated that curcumin inhibited Ti particle-induced inflammation by promoting macrophage differentiation into the M2 phenotype in a dose-dependent manner.

A murine air pouch model was used to evaluate the effects of curcumin on Ti-induced inflammation and macrophage polarization. This animal model has been widely used to research various types of inflammation due to its high sensitivity and cost efficiency (27, 28). Similar to previous studies, the injection of Ti particles into the air pouch on the backs of mice resulted in increased inflammatory cell infiltration and membrane thickness compared to the control mice (29–31). Mice treated with curcumin had a fewer number of infiltrating cells and thinner membrane formation than those injected with Ti particles alone. This phenomenon supports the anti-inflammatory effects of curcumin on Ti particle-induced inflammation. The use of the general macrophage marker F4/80 and the specific macrophage markers CCR7 (M1) and CD206 (M2) revealed that exudates retrieved from air pouches injected with Ti particles and curcumin presented a higher percentage of M2 macrophages and a lower percentage of M1 macrophages than those injected with Ti particles alone. The macrophage phenotypic changes were further verified by analyzing cytokine levels in the inflammatory exudates. Curcumin induced the secretion of lower levels of pro-inflammatory cytokines (TNF and IL-6) and higher level of anti-inflammatory cytokine IL-10. The *in vivo* results were consistent with those obtained *in vitro* and together indicate that curcumin attenuates Ti particle-induced inflammation *via* promotion of M2 macrophage differentiation and activation.

Numerous clinical and experimental reports have shown that curcumin is a safe polyphenol with a variety of pharmacological functions (15). Several recent studies have demonstrated the anti-inflammatory effects of curcumin and its therapeutic potential for various inflammatory diseases (32–34). Chin reported that curcumin could be used as a candidate for treating osteoarthritis by blocking the activation of NF-κB (35). Another recent study demonstrated that curcumin may serve as a potential therapeutic agent for the treatment of bone deterioration in rheumatoid arthritis (36). Whether curcumin have influence on the extracellular RNA should also be taken into consideration because Cabrera-Fuentes et al. reported that extracellular RNA could influence macrophage polarization state (37). To the best of our knowledge, this is the first time in which curcumin was shown to attenuate Ti particle-induced inflammation through the immunomodulation of macrophage polarization. However, the current study had several limitations that should be taken into account. First, commercially available Ti particles were used rather than other types of wear particles, such as alumina ceramic particles, UHMWPE particles, and cobalt chromium particles, which are also known causes of periprosthetic osteolysis (13, 25, 38). Whether the effects of curcumin on these types of wear particle-induced inflammatory responses are similar remains unclear. Second, the air pouch model only mimics acute inflammation of soft tissues in a very short time. Thus, a larger animal model is required to test the long-term effectiveness of curcumin on the downstream events of osteoclastogenesis and osteolysis. Third, a better drug administration method should be developed out of concern that repeated injection of curcumin may induce side effects in a clinical situation.

In conclusion, this is the first *in vitro* and *in vivo* study to show that treatment with curcumin effectively inhibits Ti particle-induced inflammation. The modulation of macrophages from M1 to M2 polarization with a shift toward macrophage-specific marker expression and cytokine secretion profiles may contribute to its anti-inflammatory properties. These results suggest that curcumin may be considered a promising candidate in the prevention and treatment of wear particle-induced osteolysis.

## Author Contributions

XZ designed the study. BL and YH performed the study and contributed equally to this work. BL drafted the manuscript, YH performed statistical analysis. YZ, MC, HQ, TC, QW, and XP helped revise the manuscript. All authors read and approved the final manuscript.

## Conflict of Interest Statement

The authors declare that the research was conducted in the absence of any commercial or financial relationships that could be construed as a potential conflict of interest.
