# Supplementary material for: Curcumin Attenuates Titanium Particle-Induced Inflammation by Regulating Macrophage Polarization In Vitro and In Vivo
Source: Front Immunol. 2017 Jan 31;8:55. doi: 10.3389/fimmu.2017.00055 (PMC5281580; doi:10.3389/fimmu.2017.00055)

Part 1


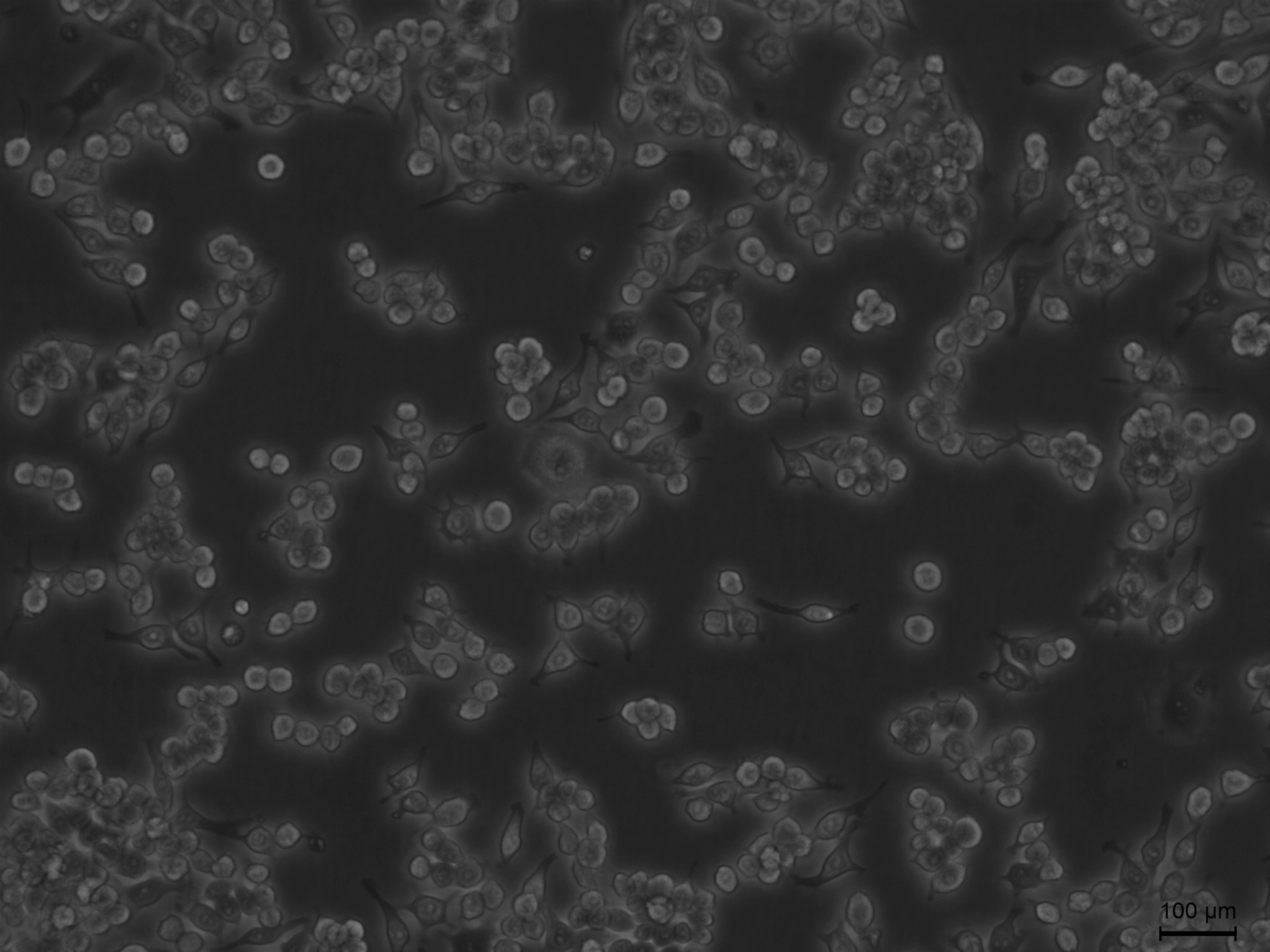


Part 2


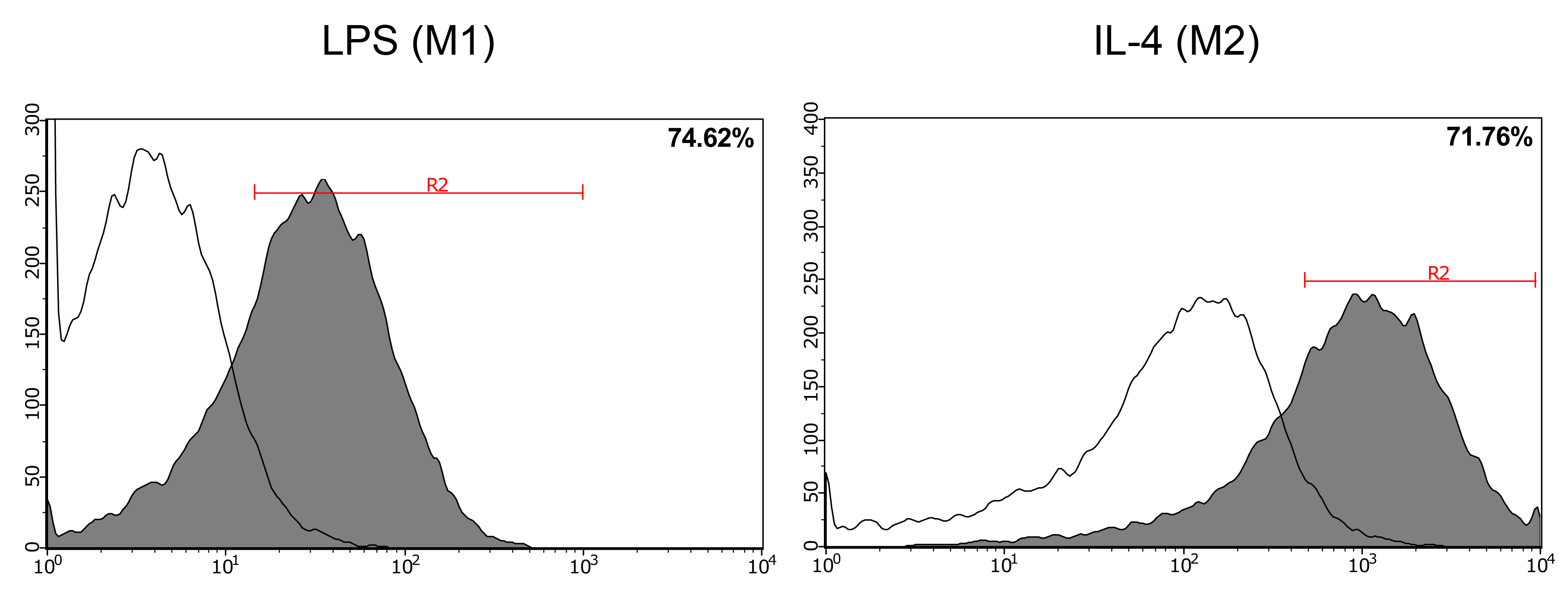


Part 3

Day 1-CCR7-Blank


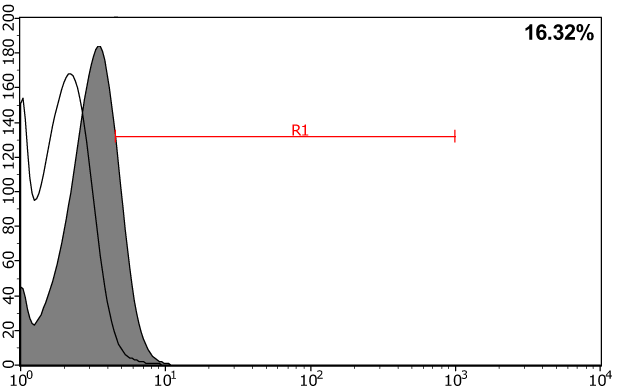


Day1-CCR7-Ti


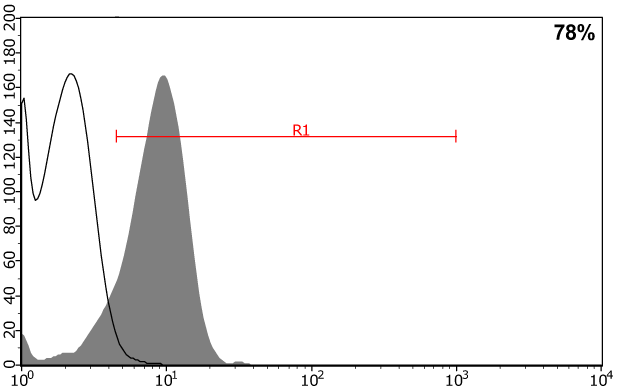


Day 1-CCR7-Ti+cur6.25


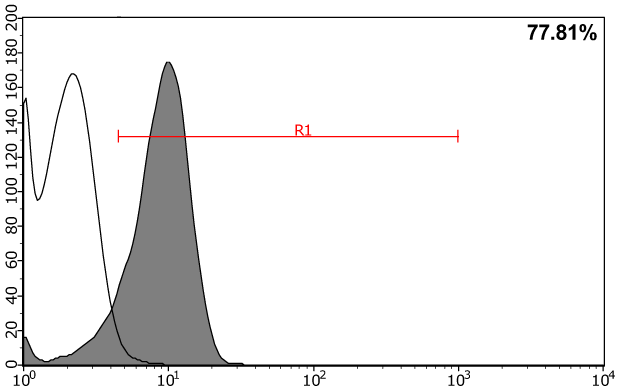


Day 1-CCR7-Ti+cur25


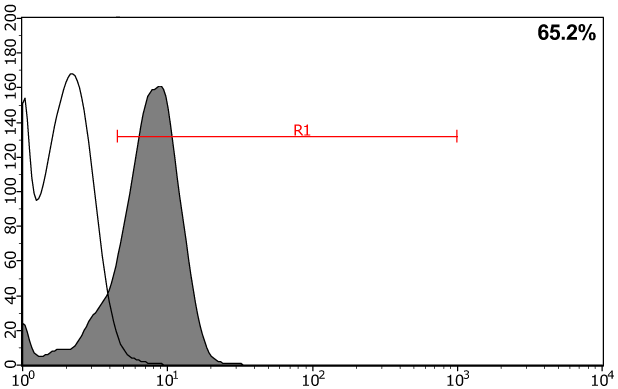


Day 1-CD206-blank


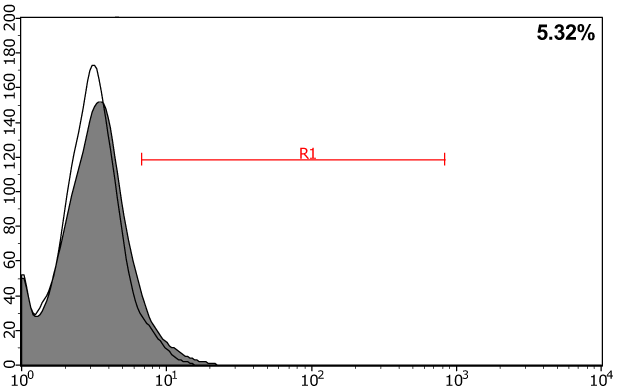


Day 1-CD206-Ti


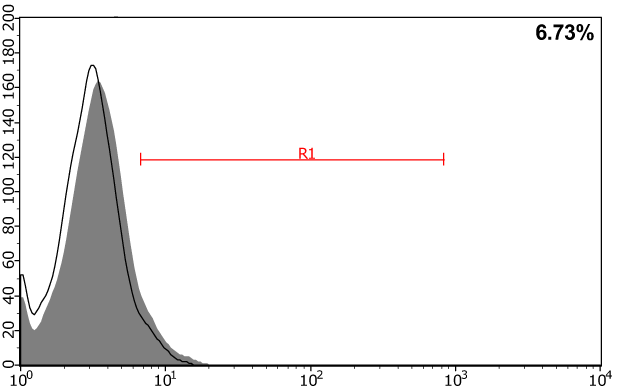


Day 1-CD206-Ti+cur6.25


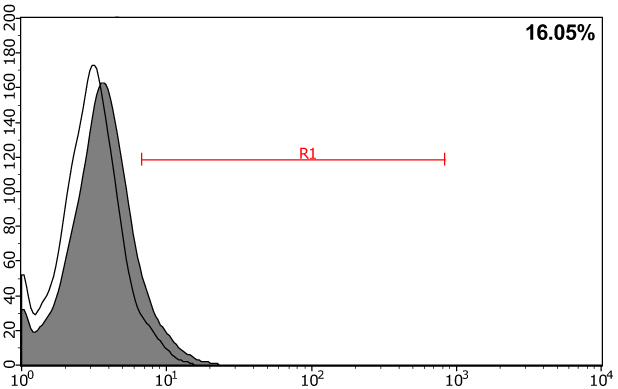


Day 1-CD206-Ti+cur25


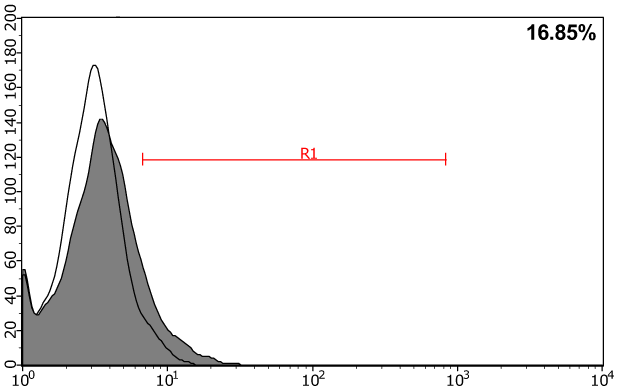

Supplement: Supplementary file 1 [file Data_Sheet_1.DOC]
